# *Toxoplasma gondii* microneme protein MIC3 induces macrophage TNF-α production and Ly6C expression via TLR11/MyD88 pathway

**DOI:** 10.1371/journal.pntd.0011105

**Published:** 2023-02-02

**Authors:** Jingfan Qiu, Yanci Xie, Chenlu Shao, Tianye Shao, Min Qin, Rong Zhang, Xinjian Liu, Zhipeng Xu, Yong Wang

**Affiliations:** Key Laboratory of Pathogen Biology of Jiangsu Province, Department of Pathogen Biology, Nanjing Medical University, Nanjing, Jiangsu, China; Tulane University, UNITED STATES

## Abstract

*Toxoplasma gondii* is the most successful parasite worldwide. It is of great interest to understand how *T*. *gondii* induce different immune responses in different hosts. In this study, we found that a peptide of *T*. *gondii* microneme protein MIC3 induced TNF-α production, NF-κB phosphorylation, *iNOS* transcription and Ly6C expression in mouse macrophage RAW264.7 cells. MyD88 inhibition, small interfering RNA against *Tlr11* and CRISPR/Cas9-mediated knock-out of *Tlr11* all reduced MIC3-induced TNF-α production, NF-κB phosphorylation, *iNOS* transcription and Ly6C expression. Additionally, we determined the location of MIC3 peptide in mouse macrophages using immunofluorescence. MIC3 could both adhere to the cell membrane of mouse macrophages and enter the cells. These results suggest that MIC3 triggered the immune responses in mouse macrophages via TLR11/MyD88/NF-κB pathway. It is known that human macrophages lacking TLR11. We predicted that the immune responses induced by MIC3 in human macrophages were significantly different from those in mouse macrophages. As expected, MIC3 peptide failed to induce TNF-α expression, *iNOS* expression and NF-κB phosphorylation in human THP-1 derived macrophages. MIC3 induced macrophage immune responses via TLR11. Intriguingly, the amino acid sequence of MIC3 is completely different from the well-known TLR11 ligand profilin, which generates a potent IL-12p40, TNF-α and IL-6 response. In marked contrast to profilin, MIC3 could not induce IL-12p40 expression in both mouse RAW264.7 cells and human THP-1 derived macrophages. Furthermore, the simulated tertiary structure of MIC3 peptide shows poor similarity with the crystal structure of profilin, suggesting that MIC3 might be a different ligand from profilin. These findings about MIC3 and TLR11 will provide us with important insights into the pathogenesis of toxoplasmosis and coevolution during host-parasite interaction.

## Author summary

To be the most successful parasite worldwide, *Toxoplasma gondii* develops fascinating ways to coexist with its host and fulfil its life cycle. After infection with *T*. *gondii*, the survival time of mice is short, while human beings can coexist with *T*. *gondii* for lifetime. Remarkably, in this study we found that a short peptide from *T*. *gondii* microneme protein MIC3, which was known to be the circulating antigen after *T*. *gondii* infection, was sufficient to trigger the TLR11/MyD88/NF-κB pathway and induced macrophage TNF-α production, NF-κB phosphorylation, *iNOS* transcription and Ly6C expression in mouse macrophages. Human macrophages lacking TLR11 failed to induce such inflammatory reaction in response to MIC3. Therefore, we speculate that the TLR11-driven deleterious inflammatory immune responses impede the long-term coexistence of host and *T*. *gondii*, which might provide the evolutionary pressures for TLR11 expression. Additionally, the amino acid sequence, simulated tertiary structure and triggered immune responses of MIC3 are completely different from the well-known TLR11 ligand profilin. All together, these findings propose MIC3 as a potential different ligand from profilin, and what we learn about MIC3 and TLR11 provides important insights into the evolutionary roles of *T*. *gondii* and other TLR11 sensing pathogens.

## Introduction

Being able to infect and multiply in all warm-blooded animals and humans, *Toxoplasma gondii* is known to be one of the most successful parasites worldwide [1]. A century after its discovery, approximately one-third of the world’s population is still infected with *T*. *gondii* [1–4]. Healthy people are able to control *T*. *gondii* infection, while individuals whose immune systems are compromised are at risk for developing fatal symptoms [5]. Additionally, *T*. *gondii* infection during pregnancy can cause adverse pregnancy outcomes. Mouse model has been developed to study *T*. *gondii* infection [6]. This is particularly because mouse is a natural intermediate host for *T*. *gondii* and likely play an important role in the life cycle of this parasite. However, different from humans, mice are highly susceptible to *T*. *gondii*. It has been reported that not only the most virulent type I strains, but also less virulent type II and type III strains of *T*. *gondii* are capable of killing immunocompetent mice after infection [7].

*T*. *gondii* is highly evolved to form a well-coordinated system. For invasion into hosts, it excretes and secretes a series of proteins from organelles, including microneme proteins (MIC), rhoptry proteins (ROP), and dense granule antigens (GRA). Among these, MICs are secreted by the microneme from the apex of *T*. *gondii*, and facilitate adhesion on the cell membrane of hosts. Thus, MICs play an important role in the recognition, adhesion and invasion of host cells [8]. As typically MIC proteins, MIC3 contains 5 partially overlapped epidermal growth factor (EGF)-like domains and a chitin binding-like (CBL) domain [9]. Thereinto, the CBL domain is associated with the adhesion characteristics of MIC3.

One of the major attributes that distinguishes parasites from other pathogens is that parasites evolve fascinating ways to coexist with their hosts for a long time. *T*. *gondii* is among the most creative organisms, developing marvelous ways to modulate the immune systems of hosts [10–12]. During recent years, it has been found that MIC3 not only plays an important role in the recognition, adhesion and invasion of host cells, but also has strong immunoreactivity. MIC3 is expressed in all stages of the *T*. *gondii* life cycle, including the tachyzoite, bradyzoite, and sporozoite stages [9]. Several studies have indicated that MIC3 could be used as a diagnostic marker or vaccine candidate molecule for toxoplasmosis [9]. Our previous study has found that MIC3 is more abundant in *T*. *gondii* RH strain than in *T*. *gondii* less virulent TgCtwh3 strain [13]. A short sequence of 73 amino acids of MIC3, which is known to be immunoreactive with sera of *T*. *gondii*-infected individuals and contains the EGF-like domains [14], exerts pro-inflammatory effects on macrophages. It could evoke a TNF-α secretory response and induce macrophage M1 polarization [13]. However, the receptor for MIC3 and the signaling pathways it modulates in macrophages are far from being complete for our knowledge. Thus, an in-depth study of MIC3 is in great need.

As a parasite-derived molecule, it is important to figure out how MIC3 could be recognized by host. Among several classes of innate immune sensors, Toll-like receptors (TLRs) recognize a variety of pathogen-associated molecular patterns (PAMPs) from almost all kinds of pathogens, including bacteria, viruses, fungi and parasites [15]. Among them, TLR11 is one of the most mysterious TLRs. For a long time, people did not know the ligand for TLR11, until 2005 a profilin-like protein from *T*. *gondii* has been demonstrated to be the ligand for TLR11 [16,17]. TLR11 is expressed in abundance in mice. However, the gene encoding TLR11 contains at least one clear-cut stop codon in human genome, which prevents the expression of TLR11 in humans [18]. In this study, we analyzed MIC3-induced immune responses and the signaling pathways in mouse and human macrophages. We found that MIC3 could govern the *T*. *gondii*-induced TNF-α production via TLR11/MyD88/NF-κB pathway in mouse macrophages. However, in human macrophages, MIC3 stimulated significantly different immune responses, which might contribute to different susceptibility of mice and humans to *T*. *gondii*. We believe that what we have learned about MIC3 and TLR11 will provide important insights into the pathogenesis of toxoplasmosis and evolutionary roles of *T*. *gondii* and other TLR11 sensing pathogens.

## Methods

### MIC3 peptide

The MIC3 peptide (amino acids 234–306) used in this study is partial of *T*. *gondii* MIC3 protein. It has the EGF-like domain IV and part of the EGF-like domain V, and contains human B and T cell epitopes [19–21]. It contains 73 amino acids and is fused to a glutathione-*S*-transferase tag. Its amino acid sequence is “RTGCHAFRENCSPGRCIDDASHENGYTCECPTGYSREVTSKAEESCVEGVEVTLAEKCEKEFGISASSCKCDN”. This sequence is highly homologous (with 100% identities) among different *T*. *gondii* strains, including Type I RH-88 and GT1 strain, Type II ME49 strain, and Type III VEG strain. This MIC3 peptide bought from Abcam (Cambridge, UK) was prepared from an *Escherichia coli* expression system and purified by a proprietary chromatographic technique. Endotoxin was removed using AffinityPak Detoxi-Gel Endotoxin Removing Gel (Thermo, Fairlawn, OH, USA). The OVA (Sigma-Aldrich, St. Louis, USA) was used as a negative control for the antigen stimulation experiments. Endotoxin levels of MIC3 and OVA were determined by a Limulus assay (Xiamen Limulus Reagent Co. Ltd., Xiamen, China) and were lower than 0.1 EU/mL.

### Cell culture

RAW264.7 cells (mouse macrophage cell line) were cultured in 6-well cell culture plates (1×10^6^ cells per well) and maintained in Dulbecco’s Modified Eagle’s Medium (DMEM) (Gibco BLR, Gaithersburg, MD, USA) supplemented with 10% fetal bovine serum (FBS) (Gemini, West Sacramento, CA, USA) at 37°C. After overnight adherence, the culture medium was changed to serum-free DMEM for starvation. After 6 h starvation, the culture medium was replaced with complete DMEM (with 10% FBS). Then 4 μg/ml OVA, 4 μg/ml MIC3 and 1 μg/ml LPS (Sigma-Aldrich) was added into the culture medium respectively. Cells were harvested after 24 h for further analysis. The supernatants were also collected for cytokine determination.

RAW264.7 cell line stably expressing Cas9 (RAW264.7-Cas9) was purchased from Ubigene (Guangzhou, Guangdong, China). RAW264.7-Cas9 cells were maintained in DMEM supplemented with 10% FBS.

THP-1 cells (human monocytic cell line) were cultured in 6-well cell culture plates (1×10^6^ cells per well) and maintained in complete RPMI 1640 medium at 37°C. For differentiation to a macrophage phenotype, THP-1 cells were treated with 10 ng/ml phorbol 12-myristate 13-acetate (PMA, Sigma-Aldrich) for 72 h, followed by a recovery period of 24 h in complete RPMI 1640 medium in the absence of PMA. A macrophage-like phenotype (cell adhesion and spreading) was observed using the Primovert Microscope (Zeiss, Germany). Then 4 μg/ml OVA, 4 μg/ml MIC3 (Abcam) and 1 μg/ml LPS were added into the culture medium respectively. Cells were harvested after 24 h for qRT-PCR analysis and Western blotting. The supernatants were collected for cytokine determination.

### ELISA

Levels of mouse TNF-α, IL-6, IL-10 and IL-12p40 were respectively measured by Mouse TNF-α, IL-6, IL-10 and IL-12p40 ELISA Kit according to the instructions of the manufacturer (Dakewe, Shenzhen, China). Levels of human TNF-α, IL-6, IL-10 and IL-12p40 in the supernatant of cell cultures were also detected by human TNF-α, IL-6, IL-10 and IL-12p40 ELISA Kit (Dakewe), respectively. All data are presented as the means ± S.D. Statistical tests to generate *P*-values are indicated in the corresponding figure legends.

### qRT-PCR

The mRNA levels of iNOS, Arg-1, IL-6, IL-10 and TNF-α in RAW264.7 and THP-1 derived macrophages were determined by real-time qRT-PCR. Total RNA of RAW264.7 cells or THP-1 derived macrophages was extracted with TRIzol reagent (Invitrogen, San Diego, CA, USA). First-strand cDNA was synthesized from 500 ng total RNA using PrimeScript RT Master Mix (Takara, Otsu, Shiga, Japan) in Veriti 96-Well Thermal Cycler (Thermo Fisher Scientific, Waltham, MA, USA). Gene-specific primers for the mouse and human genes of GAPDH, iNOS, Arg-1, IL-6, IL-10 and TNF-α were listed in S1 Table. qRT-PCR was performed using FastStart Essential DNA Green Master (Roche, Basel, Switzerland) in LightCycler 96 Instrument (Roche). The threshold cycle and melting curves were measured automatically. Data have been shown as the means ± S.D. GAPDH served as the internal control. The relative expression level of each gene was calculated using the 2^-(△△Ct)^ method.

### Western blotting

Cells were washed twice with PBS and lysed in RIPA Lysis Buffer (Millipore, Billerica, MA, USA) on ice for 10 min. After spin at 12,000 rpm for 15 min at 4°C, the supernatants were collected. The concentrations of proteins in supernatants were determined by BCA assay. SDS-PAGE sample loading buffer (Biyuntian, Shanghai, China) was added into the same amount of protein from different samples and boiled for 5 min. The proteins were separated by SDS-PAGE gel and then processed for immunoblotting. SDS-PAGE gel was transferred to PVDF membrane (Millipore, Billerica, MA, USA) followed by being blocked in TBST with 5% non-fat dry milk. Antibodies were used as follows: phospho-NF-κB p65 (Ser536) (93H1) Rabbit mAb (1:1000, Cell Signaling Technology) and HRP-conjugated goat anti-rabbit IgG secondary antibody (1:2000, Abcam). Protein bands were detected by Western Chemiluminescent HRP Substrate (ECL) (Millipore) and visualized by ChemiDoc Touch Imaging System (Bio-Rad, Hercules, CA, USA).

### Flow cytometry

To evaluate Ly6C expression on RAW264.7 cells, the cells were collected and incubated with anti-CD16/32 blocking antibody (eBioscience) at 4°C for 15 min. Then the cells were stained with PE-conjugated antibody against mouse F4/80, APC-conjugated antibody against mouse CD11b and PE-Cy7 conjugated antibody against mouse Ly6C (all from BioLegend). After incubated at 4°C in the dark for 30 min, the cells were washed twice with staining buffer at room temperature. Then the samples were evaluated using a FACSVerse flow cytometer (BD Biosciences, San Jose, CA, USA) and analyzed with FlowJo (Tree Star, Ashland, OR, USA).

### MyD88 inhibition experiments

To inhibit the activity of MyD88, RAW264.7 cells were preincubated with 20 μg/ml MyD88 inhibitor ST2825 (Apexbio, Houston, TX, USA) for 3 h at 37°C. Then cells were stimulated with OVA (4 μg/ml), MIC3 (4 μg/ml) and LPS (1 μg/ml) respectively for 24 h. After 24 h, RAW264.7 cells were collected for qRT-PCR analysis and Western blotting. The supernatants of cell culture were also collected for TNF-α determination using ELISA.

### Transfection with siRNA

RAW264.7 cells were cultured at a density of 2×10^5^ cells/well containing Opti-MEM medium in a 12-well plate. Small interfering RNA (siRNA) targeting Tlr11 (sc-61694) and control siRNA targeting a scrambled sequence (sc-37007) were purchased from Santa Cruz Biotechnology. RAW264.7 cells in each well were transfected with 120 nM siRNA using 6 μl Lipo2000 (Thermo fisher) according to the manufacturer’s instructions. The *Tlr11* knock-down efficiency was evaluated by qRT-PCR 24 h after siRNA transfection, and the mRNA levels of *Tlr11* and *Gapdh* were measured. Primers for *Tlr11* (Tlr11-qPCR-F and Tlr11-qPCR-R) were listed in S1 Table. After 24 h transfection, the cells were treated with 4 μg/ml OVA and MIC3 respectively for another 24 h. Then the knock-down efficiency was further assessed. Both the cells and supernatants were collected for further experiments. TNF-α production, *iNOS* and *Arg-1* transcription, Ly6C expression and NF-κB phosphorylation of RAW264.7 cells were analyzed after the knock-down of *Tlr11*.

### Generation of *Tlr11* knock-out cells via CRISPR/Cas9-mediated gene editing

To create a mouse *Tlr11* knock-out model in RAW264.7 cell line using CRISPR/Cas9 technology, a GFP expression vector containing two *Tlr11*-specific gRNAs (gRNA1: CTGGTGAGCCTTACCTTGACTGG and gRNA2: GTACAACCAGTGTCACATCTAGG) was constructed (YKO-RP009-mTlr11). Then the YKO-RP009-mTlr11 plasmids were introduced into RAW264.7-Cas9 cells using Amaxa 4D-Nucleofector kit (Lonza, Cologne, Germany), which could generate a large deletion in exon 2 of *Tlr11*. Two days post nucleofection, GFP^+^ cells were sorted by flow cytometry and single clones were seeded in 96-well plates. Subsequently, single-cell clones were expanded and genotyped using PCR with primers TLR11-F and TLR11-R. The PCR products of clones with predicted *Tlr11*-deletion were confirmed via Sanger sequencing. After sequencing, the *Tlr11*^-/-^ clones were further verified with qRT-PCR using primers Tlr11-qPCR-N-F and Tlr11-qPCR-N-R, which were designed inside the deletion region of *Tlr11*.

### Immunofluorescence assays

For immunofluorescence analyses, cells were plated in 20 mm Glass Bottom Cell Culture Dish for laser confocal (NEST, Wuxi, China), washed three times with staining buffer (1% BSA in PBS), and then fixed in fresh 4% paraformaldehyde-PBS for 30 min at 37°C. After being washed in staining buffer, the cells were blocked with 5% BSA in PBS at 37°C for 30 min. To characterize the cellular localization of MIC3, cells were washed and incubated with primary anti-MIC3 mouse monoclonal antibody (GeneTex, Irvine, CA, USA) and PE-conjugated antibody against mouse F4/80 overnight at 4°C. After being washed, cells were incubated with secondary Alexa Fluor 488 AffiniPure Goat Anti-Mouse IgG antibody (Yeason, Shanghai, China) at 4°C in dark for 30 min. To determine the expression of p-NF-κB, the phospho-NF-κB p65 (Ser536) (93H1) Rabbit mAb (Cell Signaling Technology) and secondary antibody Alexa Fluor 488 Goat Anti-Rabbit IgG H&L (Abcam) were used and followed the same procedure as described previously. DAPI (Servicebio, Wuhan, China) was used to mark the nuclei at 4°C in dark for 10 min. Slides were mounted with Fluoromount-G reagent (Invitrogen) and kept in the dark until viewing. The slides were examined under a laser confocal microscopy CarlZeiss LSM710 (Carl Zeiss, Germany).

### Statistical analyses

SPSS software was used to determine the statistical significance of differences in the means of experimental groups. Data of two groups were analyzed for statistical significance with Student’s *t*-test. Multiple comparisons were made by one-way ANOVA.

## Results

### MIC3 induced TNF-α expression in mouse macrophages by activating NF-κB

**Fig 1:**
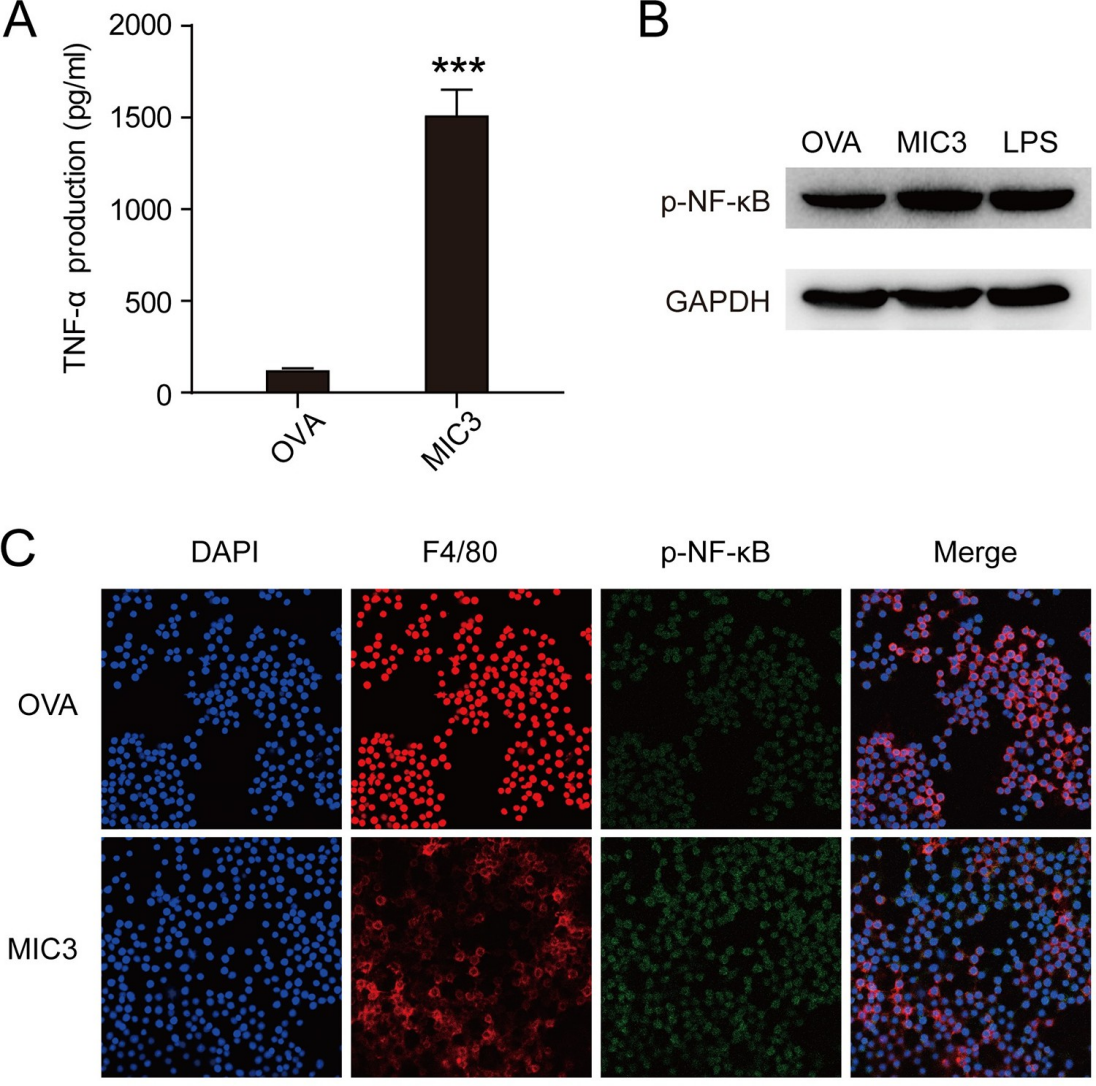
MIC3 induced TNF-α expression and NF-κB p65 phosphorylation in RAW264.7 cells. **(A)** The levels of TNF-α secreted by RAW264.7 cells after 24 h-treatment of 4 μg/ml MIC3 and OVA. Data are expressed as the means ± S.D. of four independent samples for each group. Significance was determined by the two-tailed Student’s *t*-test. ***, *P*<0.001. **(B)** NF-κB p65 phosphorylation of macrophages after the treatment of MIC3. The NF-κB p65 phosphorylation of RAW264.7 cells after 24 h-treatment of 4 μg/ml MIC3, 4 μg/ml OVA and 1 μg/ml LPS was detected by Western blotting. **(C)** The NF-κB p65 phosphorylation of RAW264.7 cells after 24 h-treatment of 4 μg/ml MIC3 and 4 μg/ml OVA was evaluated by immunofluorescence assay.

Our previous study found that MIC3 from *T*. *gondii* ESAs was a dominant factor in the induction of TNF-α expression [13]. In this study, we measured the levels of TNF-α secreted by mouse macrophage RAW264.7 cells after the treatment of MIC3 peptide. The MIC3 peptide contains 73 amino acids and is known to be immunoreactive with sera of *T*. *gondii*-infected individuals [14]. After stimulation with 4 μg/ml OVA or MIC3 peptide for 24 h, the levels of TNF-α in the MIC3 group were significantly higher than those in OVA control (130.77 ± 1.40 pg/ml vs. 1520.20 ± 132.09 pg/ml, *P* < 0.001) (Fig 1A). Thus, MIC3 peptide could induce pro-inflammatory cytokine TNF-α expression in mouse macrophages.

It was reported that after LPS stimulation, phosphorylated NF-κB p65 could bind to the TNF promoter and induce TNF-α expression [22,23]. In this study, Western blotting results showed that MIC3 potently phosphorylated p65 in the same level as LPS (Fig 1B), indicating MIC3-induced TNF-α expression was associated with phosphorylation of NF-κB p65. Then, MIC3-treated RAW264.7 cells were fixed and stained with anti-F4/80 and anti-phospho-NF-κB p65 immunofluorescence antibodies respectively. The immunofluorescence assay showed that the expression of phosphorylated NF-κB p65 in the MIC3-treated cells was significantly higher than that in OVA control group (Fig 1C). Phosphorylated p65 was evident in the RAW264.7 cells after 24 h-MIC3 stimulation. In sum, MIC3 enhanced phosphorylation of NF-κB p65 and induced TNF-α expression.

### MIC3 enhanced the expression of inflammatory markers in macrophages

**Fig 2:**
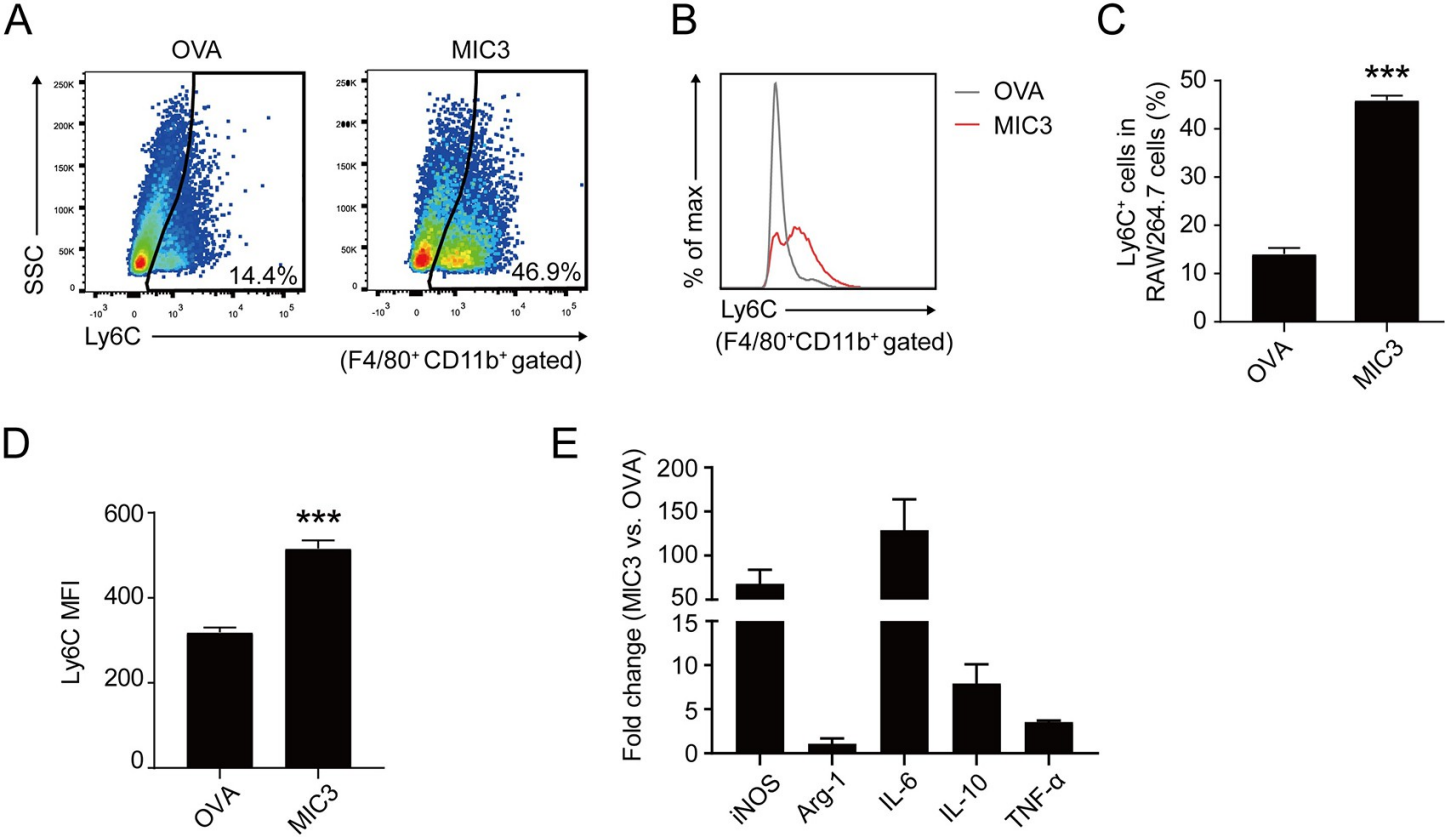
The expression levels of inflammation markers in RAW264.7 cells after 24 h-treatment of MIC3. **(A)** Ly6C expression in RAW264.7 cells. After 24 h-treatment of 4 μg/ml OVA and 4 μg/ml MIC3, Ly6C expression on F4/80^+^CD11b^+^ gated RAW264.7 cells were evaluated by flow cytometry. One representative of three independent experiments is shown. **(B)** Overlay of representative histograms showing Ly6C expression in RAW264.7 cells treated with 4 μg/ml OVA and 4 μg/ml MIC3. **(C)** The percentage of Ly6C^+^ cells in F4/80^+^CD11b^+^ gated RAW264.7 cells. Data are expressed as the means ± S.D. (n = 3 independent experiments). Significance was determined by the two-tailed Student’s *t*-test. ***, *P* < 0.001. **(D)** The mean fluorescence intensity (MFI) of Ly6C expression in F4/80^+^CD11b^+^ gated RAW264.7 cells. Data are expressed as the means ± S.D. of three independent samples for each group. Significance was determined by the two-tailed Student’s *t*-test. ***, *P* < 0.001. **(E)** The mRNA levels of inflammatory genes after 24 h-treatment of 4 μg/ml MIC3 in RAW264.7 cells. Data are expressed as the means ± S.D. of four independent samples for each group.

Pathogenic TNF mainly originates from M1-type Ly6C^+^ macrophages to exert antiparasitic function. Thus, we used flow cytometry to test the effect of MIC3 on Ly6C^+^ cells in macrophages. The frequency of Ly6C^+^ cells in F4/80^+^CD11b^+^ macrophages increased about 3-fold in the MIC3-treated group compared with that in the OVA-treated control group (*P* < 0.001, Fig 2A–2C). Similar patterns were observed when Ly6C expression was analyzed by mean fluorescence intensity (MFI). MIC3 also increased the MFI of Ly6C expression in RAW264.7 cells (*P* < 0.001, Fig 2D).

Furthermore, MIC3 upregulated the mRNA levels of *iNOS* in mouse macrophages, which is an M1-polarization marker (Fig 2E). However, the mRNA levels of *Arg-1*, which is an M2-polarization marker, did not exhibit any significant change after MIC3 treatment. Additionally, MIC3 increased the mRNA levels of *Il-6* and *Il-10*. These results confirmed that MIC3 induced a proinflammation status of macrophages.

### Involvement of MyD88 in MIC3-induced innate immune response

**Fig 3:**
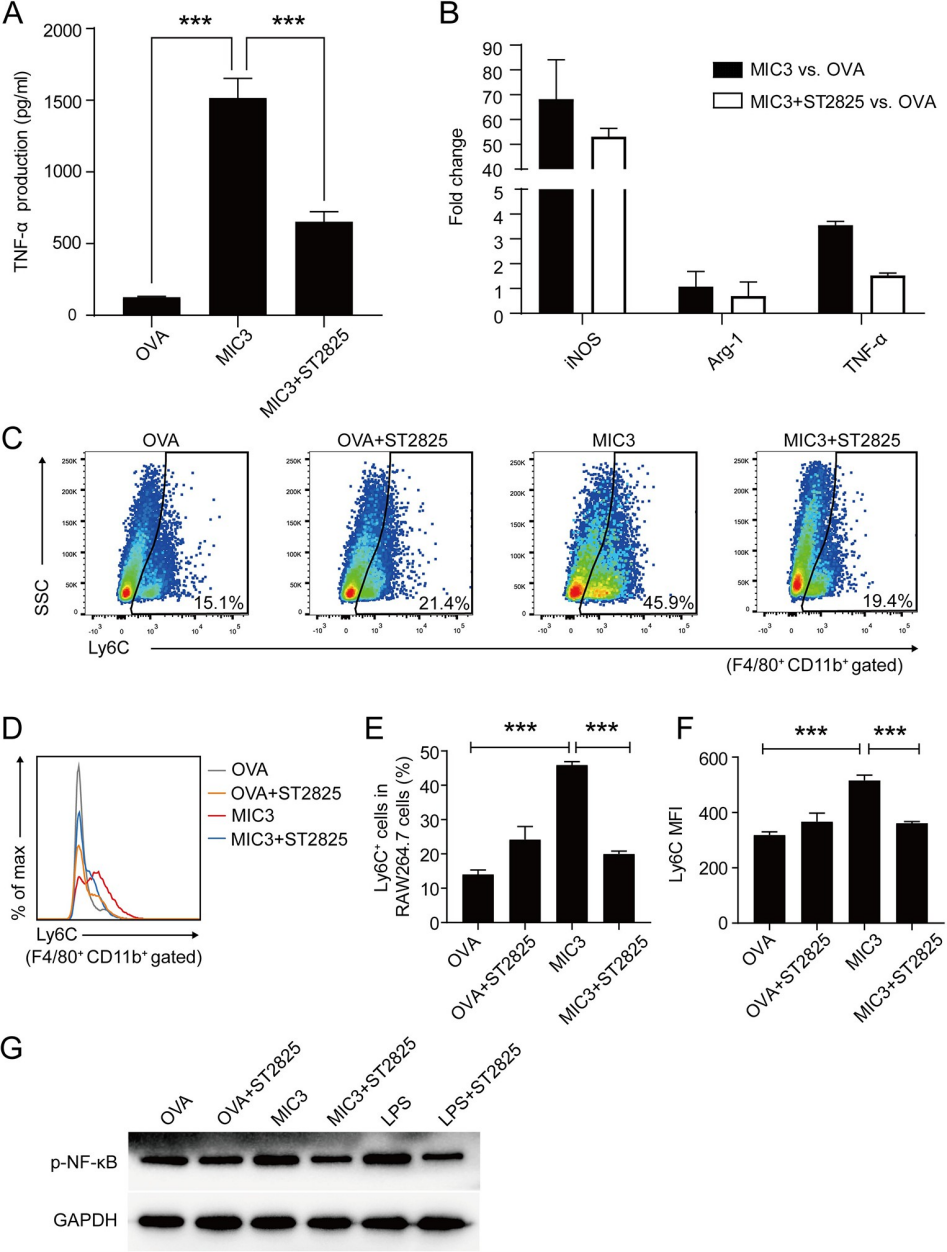
The role of MyD88 in inflammatory immune response induced by MIC3 in RAW264.7 cells. **(A)** The levels of TNF-α secreted by RAW264.7 cells after 24 h-treatment of 4 μg/ml MIC3 and OVA. To inhibit the activity of MyD88, RAW264.7 cells in group MIC3+ST2825 were preincubated with 20 μg/ml MyD88 inhibitor ST2825 for 3 h. The secretion of TNF-α was analyzed by ELISA. Each bar indicates the mean value ± S.D. (n = 4 independent replicates). Significance was analyzed using one-way ANOVA. ***, *P* < 0.001. **(B)** The mRNA levels of *Tnf-α* and polarization-related genes after the pretreatment of MyD88 inhibitor ST2825 and 24 h-treatment of MIC3 in RAW264.7 cells. Data are expressed as the means ± S.D. (n = 4 independent replicates). 1 = no change. **(C)** Ly6C expression in MyD88-inhibited RAW264.7 cells. Ly6C expression in RAW264.7 cells preincubated with 20 μg/ml MyD88 inhibitor ST2825 and treated with 4 μg/ml OVA and 4 μg/ml MIC3 were evaluated by flow cytometry. One representative of three independent experiments is shown. **(D)** Overlay of representative histograms showing Ly6C expression in MyD88-inhibited RAW264.7 cells treated with 4 μg/ml OVA and 4 μg/ml MIC3. **(E)** The percentage of Ly6C^+^ cells in F4/80^+^CD11b^+^ gated RAW264.7 cells. Data are expressed as the means ± S.D. (n = 3 independent experiments). Significance was analyzed using one-way ANOVA. ***, *P* < 0.001. **(F)** The MFI of Ly6C expression in F4/80^+^CD11b^+^ gated RAW264.7 cells. Each bar indicates the mean value ± S.D. (n = 3 independent experiments). Significance was analyzed using one-way ANOVA. ***, *P* < 0.001. **(G)** The effects of MyD88 inhibitor ST2825 on MIC3 and LPS-induced NF-κB p65 phosphorylation. The NF-κB p65 phosphorylation of RAW264.7 cells was evaluated by Western blotting.

Innate sensing of infection is of paramount importance for triggering host resistance to invading pathogens. Among several classes of innate immune sensors, TLRs and their adaptor protein myeloid differentiation factor 88 (MyD88) play crucial roles in initiating the innate immune responses [24,25]. To figure out whether MIC3 regulates innate immune response via MyD88-dependent signaling pathway, cells were pre-treated with ST2825, which is an inhibitor of MyD88. Then, the levels of secreted TNF-α were detected by ELISA. As shown in Fig 3A, in cells without inhibitors, the production of TNF-α significantly increased after the treatment of MIC3, compared with that in control group (*P* < 0.001). However, with the presence of inhibitors, the TNF-α production induced by MIC3 was suppressed and was significantly lower than that without inhibitors (*P* < 0.001). The results suggested that MIC3 exerted its effect on macrophages through TLR/MyD88-dependent pathway. After blocking the MyD88 adaptor, the mRNA levels of *iNOS* decreased (Fig 3B). The mRNA levels of *Tnf-α* in mouse macrophages were also down-regulated. Meanwhile, the proportion of F4/80^+^CD11b^+^Ly6C^+^ macrophages induced by MIC3 decreased significantly after blocking the MyD88 adaptor (Fig 3C–3E). Similar patterns were observed when Ly6C expression was analyzed by MFI (Fig 3F). Furthermore, the MIC3- and LPS-enhanced phosphorylation of NF-κB p65 were both suppressed by the inhibitor of MyD88 (Fig 3G). Thus, it was obvious that MIC3 regulated innate immune response of mouse macrophages via MyD88-dependent signaling pathway.

### MIC3 inducing immune responses via TLR11

**Fig 4:**
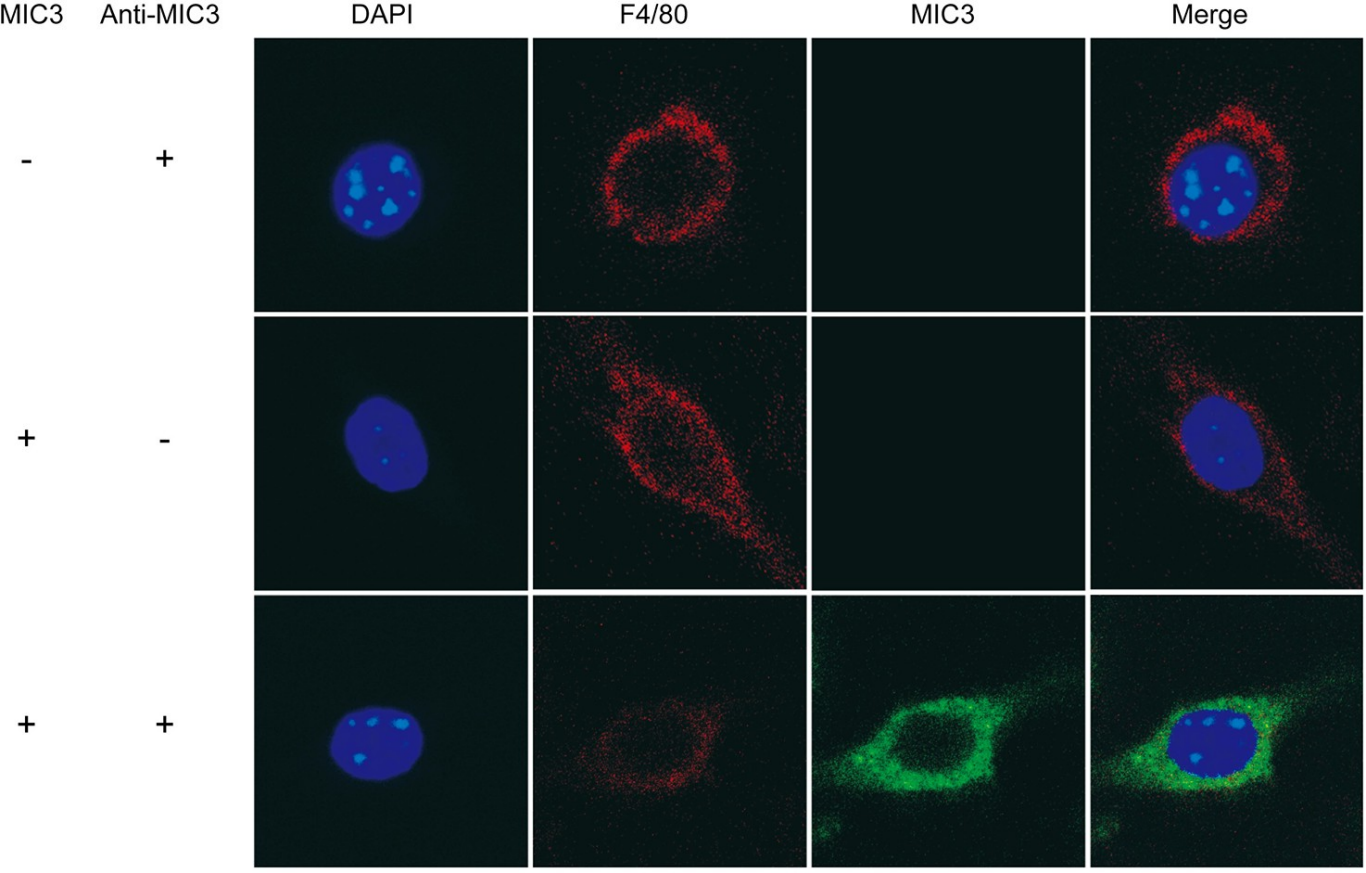
The location of MIC3 in RAW264.7 cells detected by laser confocal microscopy. To characterize the cellular localization of MIC3, cells were incubated with primary anti-MIC3 mouse monoclonal antibody (Anti-MIC3) and PE-conjugated antibody against mouse F4/80 overnight at 4°C. After being washed, cells were incubated with secondary Alexa Fluor 488 AffiniPure goat anti-mouse IgG antibody (Anti-IgG). DAPI: cell nucleus; F4/80: macrophage surface marker; MIC3: microneme protein 3 of *T*. *gondii*.

MIC3 regulated innate immune response of mouse macrophages via MyD88-dependent signaling pathway, which is a classic downstream signaling pathway of TLRs. To identify the receptor for MIC3, we first determined the location of MIC3 in mouse macrophages. We treated RAW264.7 cells with 4 μg/mL MIC3 for 4 h. The surface marker F4/80 of macrophages and MIC3 were both stained. Immunofluorescence staining showed that MIC3 could not only adhere to the cell membrane of mouse macrophages but also enter the cells (Fig 4). Since TLR11 is not only detected on the surface of cells, but also redistributes toward intracellular localization, the endolysosomes, within 1 h of profilin stimulation [26], this raises a question: is TLR11 involved in MIC3-induced immune responses?

**Fig 5:**
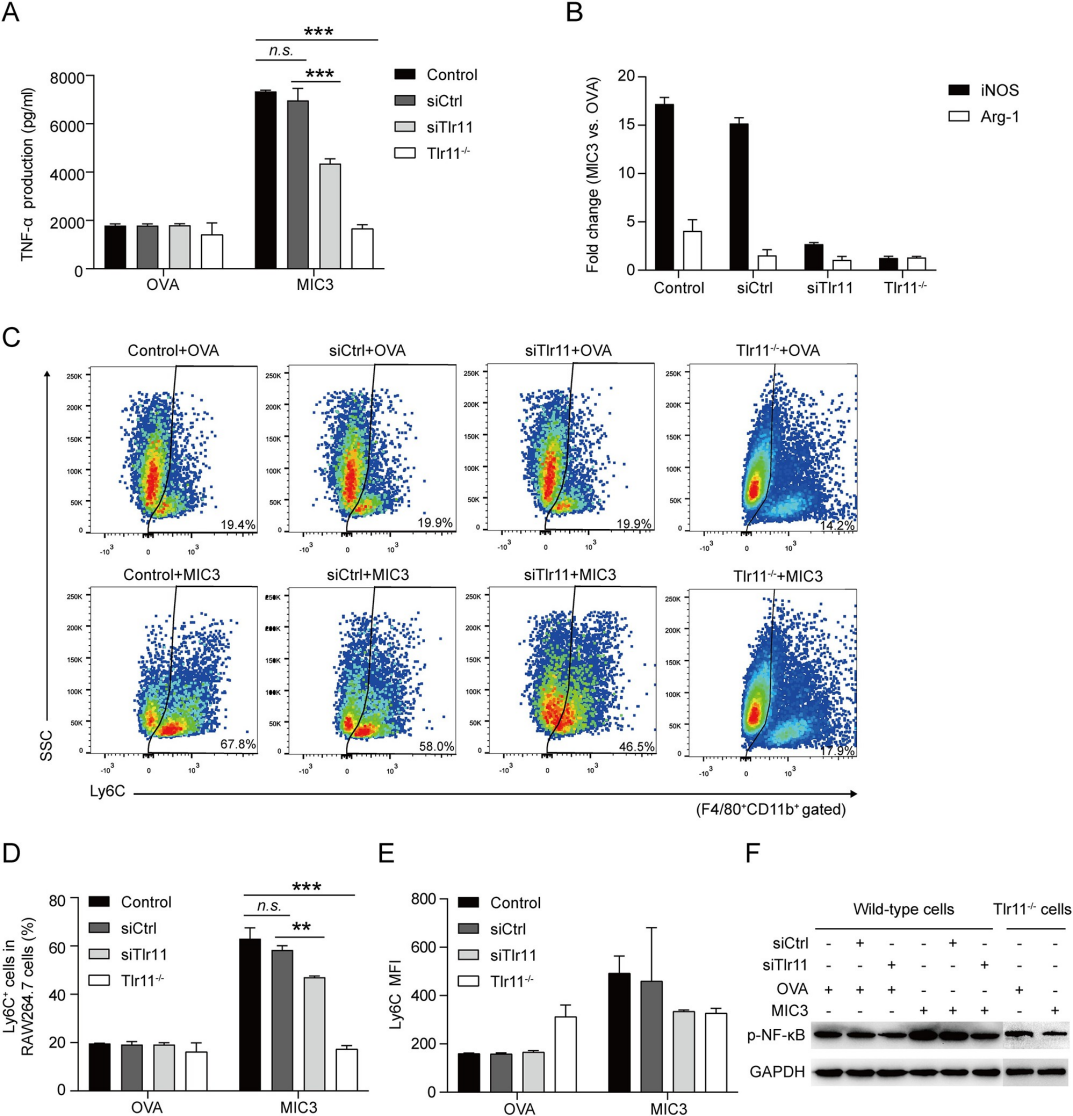
The effects of *Tlr11* knock-down and knock-out on MIC3-induced immune responses in RAW264.7 cells. **(A)** The effects of *Tlr11* knock-down and knock-out on TNF-α secretion of RAW264.7 cells after 24 h-treatment of 4 μg/ml MIC3 or OVA. To knock-down the *Tlr11*, siRNA targeting *Tlr11* (siTlr11) and a control siRNA (siCtrl) were used. CRISPR/Cas9 technology was used to create a *Tlr11* knock-out RAW264.7 cell line (*Tlr11*^*-/-*^). Each bar indicates the mean value ± S.D. (n = 3 independent experiments). Significance was analyzed using one-way ANOVA. ***, *P* < 0.001. *n*.*s*., *P* > 0.05. **(B)** The effects of *Tlr11* knock-down and knock-out on the mRNA levels of polarization-related genes (*iNOS* and *Arg-1*) after 24 h-treatment of MIC3 in RAW264.7 cells. Data are expressed as the means ± S.D. (n = 3 independent experiments). 1 = no change. **(C)** Ly6C expression in *Tlr11* knock-down and knock-out RAW264.7 cells. Ly6C expression after 24 h-treatment with 4 μg/ml OVA and 4 μg/ml MIC3 were evaluated by flow cytometry. One representative of three independent experiments is shown. **(D)** The percentage of Ly6C^+^ cells in F4/80^+^CD11b^+^ gated RAW264.7 cells. Each bar indicates the mean value ± S.D. (n = 3 independent experiments). Significance was analyzed using one-way ANOVA. ***, *P* < 0.001. **, *P* < 0.01. *n*.*s*., *P* > 0.05. **(E)** The MFI of Ly6C expression in F4/80^+^CD11b^+^ gated RAW264.7 cells. Data are expressed as the means ± S.D. (n = 3 independent experiments). **(F)** The effects of *Tlr11* knock-down and knock-out on MIC3-induced NF-κB p65 phosphorylation. The NF-κB p65 phosphorylation of RAW264.7 cells was evaluated by Western blotting.

To confirm the role of TLR11 in MIC3-induced immune responses, we both knocked-down and knocked-out *Tlr11* in RAW264.7 cells. *Tlr11* expression levels were dramatically reduced by siRNA targeting *Tlr11* (siTlr11) at 24 h and 48 h after siRNA transfection (S1 Fig). A control siRNA (siCtrl) that targets a scrambled sequence had no effect on *Tlr11* expression. *Tlr11*^*-/-*^ RAW264.7 cell line, which was generated using CRISPR/Cas9 technology, has a 243 bp deletion in exon 2 of *Tlr11* and several frame-shift mutations (S2 Fig and S2 Table). As a readout, TNF-α production, polarization marker transcription, Ly6C expression and NF-κB phosphorylation following MIC3 stimulation were analyzed. Both knock-down and knock-out of *Tlr11* significantly reduced TNF-α production by RAW264.7 cells in response to MIC3 stimulation (Fig 5A). By contrast, the siCtrl had no effect on MIC3-induced TNF-α production. After knocking down the expression of *Tlr11*, the mRNA levels of *iNOS* decreased compared with the siRNA control group (Fig 5B). As expected, *Tlr11*^*-/-*^ RAW264.7 cells did not exhibit any MIC3-induced increase at the transcriptional levels of *iNOS* and *Arg-1*. Meanwhile, the proportion of F4/80^+^CD11b^+^Ly6C^+^ macrophages induced by MIC3 all decreased remarkably after knocking down and knocking out the expression of *Tlr11* (Fig 5C and 5D). The MFI of Ly6C expression induced by MIC3 was also down-regulated after *Tlr11* knock-down (Fig 5E). Moreover, the MIC3-enhanced phosphorylation of NF-κB p65 were suppressed in siTlr11 group, while siCtrl had no effect on MIC3-induced NF-κB p65 phosphorylation (Fig 5F). Consistently, MIC3 did not up-regulate any phosphorylation of NF-κB p65 in *Tlr11*^*-/-*^ group, when compared to OVA control. Thus, it was obvious that MIC3 induced immune responses of macrophages via TLR11/MyD88 pathway and MIC3 was a potential ligand for TLR11.

### Discrepancy between mouse and human macrophages after the treatment of MIC3

**Fig 6:**
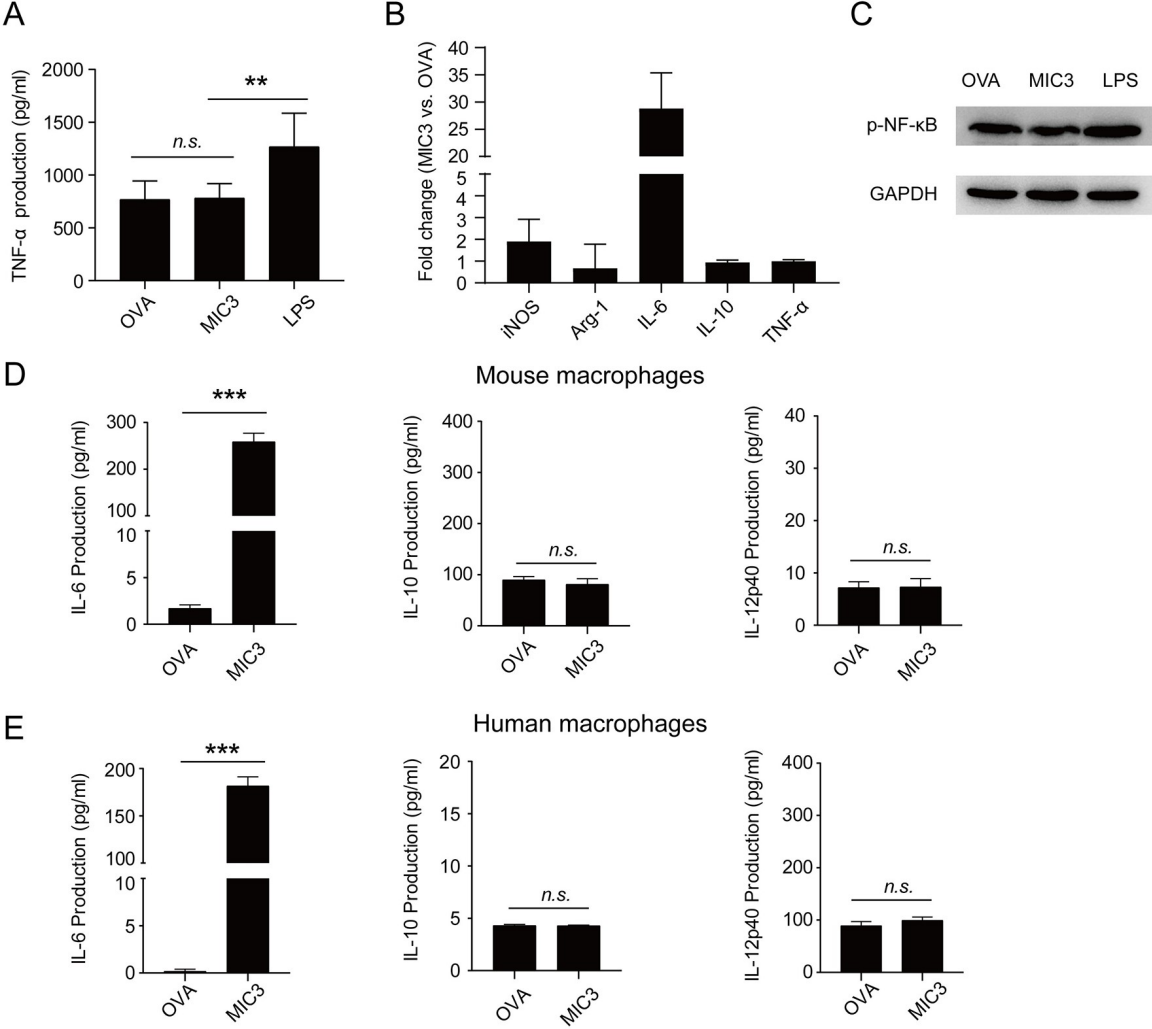
MIC3-induced innate immune responses in THP-1 derived macrophages and cytokine responses in RAW264.7 cells. **(A)** The levels of TNF-α secreted by THP-1 derived macrophages after 24 h-treatment of MIC3. THP-1 cells were incubated with PMA to differentiate into THP-1 derived macrophages. Then 4 μg/ml OVA, 4 μg/ml MIC3 and 1 μg/ml LPS were added into the culture medium respectively. The levels of TNF-α were evaluated by ELISA. Data are expressed as the means ± S.D. of six independent samples for each group. Significance was determined by one-way ANOVA. **, *P* < 0.01. *n*.*s*., *P* > 0.05. **(B)** The mRNA levels of polarization and inflammation-related genes after 24 h-treatment of MIC3 in THP-1 derived macrophages. Data are expressed as the means ± S.D. of three independent samples for each group. **(C)** NF-κB p65 phosphorylation of THP-1 derived macrophages after the treatment of MIC3. The NF-κB p65 phosphorylation of THP-1 derived macrophages after 24 h-treatment of 4 μg/ml MIC3, 4 μg/ml OVA and 1 μg/ml LPS was detected by Western blotting. **(D)** Cytokines secreted by mouse RAW264.7 cells after the treatment with MIC3. The levels of IL-6, IL-10 and IL-12p40 secreted by RAW264.7 cells after 24 h-treatment of 4 μg/ml MIC3 and OVA were detected by ELISA. Data for IL-6 and IL-12p40 are expressed as the means ± S.D. (n = 3 independent experiments). Data for IL-10 are expressed as the means ± S.D. (n = 4 independent experiments). Significance was determined by the two-tailed Student’s *t*-test. ***, *P* < 0.001. *n*.*s*., *P* > 0.05. **(E)** Cytokines secreted by THP-1 derived macrophages after the treatment with MIC3. The levels of IL-6, IL-10 and IL-12p40 secreted by THP-1 derived macrophages after 24 h-treatment of 4 μg/ml MIC3 and OVA were detected by ELISA. Data are expressed as the means ± S.D. (n = 4 independent experiments). Significance was determined by the two-tailed Student’s *t*-test. ***, *P* < 0.001. *n*.*s*., *P* > 0.05.

In the case of *T*. *gondii* infection, TLR11 generates a potent IL-12p40 and TNF-α response [17]. However, Functional TLR11 is present in mice but absent in humans [18]. In this study, we demonstrated that MIC3 induced a TNF-α response via TLR11/MyD88 pathway. Thus, we speculated that MIC3 could not induce similar immune responses in human macrophages due to the absence of TLR11. To test this hypothesis, PMA was first added into cell culture to differentiate THP-1 into THP-1 derived macrophages. Then MIC3, OVA (negative control) and LPS (positive control) were added into the culture medium, respectively. As expected, MIC3 stimulation had no effect on TNF-α production, *iNOS* transcription and NF-κB p65 phosphorylation in THP-1 derived macrophages (Fig 6A–6C). These results demonstrated that MIC3-induced immune responses in human macrophages were completely different from those in mouse macrophages.

Meanwhile, we analyzed the IL-6, IL-10 and IL-12p40 cytokine responses of MIC3 in both mouse and human macrophages. MIC3 induced the production of IL-6 and TNF-α in mouse RAW264.7 cells, but only induced the IL-6 expression in human THP-1 derived macrophages (Fig 6D and 6E). This result indicated that MIC3-induced IL-6 expression might not be associated with TLR11. Additionally, MIC3 could not induce IL-10 or IL-12p40 expression in both RAW264.7 and THP-1 derived macrophages.

It is known that the first identified TLR11 ligand, *T*. *gondii* profilin-like protein, generates a potent IL-12p40, TNF-α and IL-6 response. To analyze the homology of *T*. *gondii* MIC3 peptide and profilin-like protein, we compared the amino acid sequences of these two proteins. It has been found that amino acid sequences of MIC3 peptide and profilin-like protein show low homology (S3 Fig). K. Kucera *et al* analyzed the crystal structure of *T*. *gondii* profilin and reported that the acidic loop and β-hairpin motifs of profilin were critical for TLR11 recognition [27]. However, MIC3 shows low homology with profilin in amino acid sequences, and does not have acidic loop and β-hairpin motifs. To clarify whether MIC3 and profilin are similar in tertiary structure, SWISS-MODEL (https://swissmodel.expasy.org/) was used to simulate the tertiary structure of MIC3 peptide [28]. The simulated tertiary structure of MIC3 showed poor similarity with the crystal structure of profilin (S4 Fig). All these findings proposed that MIC3 might be a different ligand from profilin.

## Discussion

Sensing specific molecules from invading pathogens by pattern recognition receptors (PRRs) is important for the host to mount an immune response [29]. Studies have shown that TLRs known to be related to the molecules of *T*. *gondii* can be divided into two categories [30]. One is TLR2, which can recognize the small molecular glycosylphosphatidylinositol (GPI) of *T*. *gondii* [31]. The other is TLR11, which identifies profilin from *T*. *gondii* and generates a powerful NF-κB-dependent inflammatory response [17]. In this study, we found that MIC3 peptide originating from *T*. *gondii* microneme might be a potential ligand for TLR11. It could induce mouse macrophage TNF-α production and Ly6C expression via TLR11/MyD88 pathway.

As the first identified ligand for TLR11 [17], profilins are a class of small actin-binding proteins that are essential for the gliding motility of *T*. *gondii* and invasion into host cells [32]. *T*. *gondii* profilin protein is present in abundance in soluble antigen [16]. Crystal structure study of *T*. *gondii* profilin reveals a parasite-specific surface motif consisting of an acidic loop, followed by a long β-hairpin [27]. The acidic loop/β-hairpin motif is required for TLR11 recognition [27]. Protein sequence alignment reveals that the identified *T*. *gondii* profilin protein shares significant homology only with profilin genes from other apicomplexan protozoa. Therefore, TLR11 is also involved in the recognition of these parasites, such as *Neospora caninum* and the malaria parasite [16]. Additionally, TLR11 is also a receptor for flagellin FliC from human uropathogenic *Escherichia coli* and *Salmonella* [18,33]. There is an 8/13 sequence match between actin and flagellin in their N-terminal regions [34]. Following the same paradigm as all known TLR ligands, proteins have conserved features with *T*. *gondii* profilins should be potential TLR11 ligands. However, MIC3 is completely different from profilin. The molecular basis for the recognition of MIC3 by TLR11 remains unknown, and should be further studied.

*T*. *gondii*-derived molecules are potent trigger of proinflammatory cytokines, which contribute to defense against parasites and enable host survival. However, the induction of proinflammatory cytokines must be tightly regulated, because overproduction of these cytokines could cause self-damaging. Therefore, a balance of immune responses is crucial for the survival of the hosts [35]. TLR11-mediated immune responses of macrophages play multiple biological roles during *T*. *gondii* infection. The first role is to stimulate cytokine production, such as TNF-α and IL-12p40. The second mission is to regulate nitric oxide (NO)-related anti-infection activities. It is known that inflammatory cytokine TNF-α induces NO production in RAW264.7 cells, and NO could directly kill invading pathogens [36]. Thirdly, besides the responses against invading *T*. *gondii*, overproduction of inflammatory cytokines would impair host tissue [37–39]. Therefore, TLR11-driven innate inflammatory responses act as a double-edged sword. The levels of TNF-α induced by infection would be worthy of investigation in detail.

In this study, we found that MIC3 mounted TNF-α expression in mouse macrophages via TLR11/MyD88 pathway, while had no effect on TNF-α production in human macrophages. The lack of TNF-α leads to acute susceptibility to *T*. *gondii* infection. But when pathogenic TNF-producing Ly6C^+^ macrophages rise sharply, the tissue damage caused by TNF-α will aggravate. MIC3 is expressed in the tachyzoite, bradyzoite and sporozoite stages of *T*. *gondii* [9]. It is an important circulating antigen in host blood. Thus, after *T*. *gondii* infection, MIC3 and profilin interact with TLR11 and induce potent immune responses in mice. However, overexpression of proinflammatory cytokines results in a cytokine storm and even systemic inflammatory response syndrome (SIRS) in mice. In marked contrast to the murine immune system, human cells are lack of TLR11 and have different sensor system for *T*. *gondii* infection. Kevin W. Tosh et al has found that phagocytosis of live tachyzoites is required for cytokine response of human myeloid cells to *T*. *gondii* [40]. Alexandra Safronova et al has demonstrated that human cells recognize the presence of *T*. *gondii* infection by detecting the alarmin S100A11 via its receptor RAGE, which induce a potent chemokine CCL2 required for host resistance to the parasite [11].

Humans are tolerant hosts to *T*. *gondii* infection, while mice are susceptible to *T*. *gondii*. How a gene expressed in mice, but not in humans, actually relates to human occult infection? In considering this scenario, the first thought was that the gene of TLR11 did exist in the human genome. Several groups have repeated the genomic analysis and have confirmed that remnants of the human TLR11 do exist; however, the predicted mRNA has at least one clear-cut stop codon that result in failed expression of TLR11 [18]. The absent expression of TLR11 in humans is more likely the result of evolution. The prediction might be helped when genes of TLR11 from more species were analyzed. TLR11 is present in the mouse and rat genomes but not in the genomes of cat, dog, frog, chimp and fugu [16,41]. Thus, we can imagine the evolutionary pressures that cause TLR11 to downgrade to a non-coding gene in humans and certain animals. The evolutionary absence of TLR11 might be a form of protection. This study provides novel insights into the interaction of *T*. *gondii* with its host and the evolutionary roles of *T*. *gondii*.

## Supporting information

S1 Table Primers used in this study. (DOCX)

S2 Table *Ct* values of *Tlr11* in wild-type and *Tlr11^-/-^* RAW264.7 cells. (DOCX)

S1 Fig ***Tlr11* knock-down efficiency in RAW264.7 cells 24 h (A) and 48 h (B) after transfection.** *Tlr11* expression levels were knocked-down by siRNA targeting *Tlr11* (siTlr11). A control siRNA (siCtrl) that targets a scrambled sequence were used as control. 24 h after transfection, RAW264.7 cells were treated with 4 μg/ml MIC3 and OVA for another 24 h. The mRNA levels of *Tlr11* and *Gapdh* were measured 24 h and 48 h after siRNA transfection using qRT-PCR. 1 = no change. Each bar indicates the mean value ± S.D. (n = 3 independent replicates).
(TIF)

S2 Fig The sequence verification for *Tlr11* knock-out RAW264.7 cells. The PCR products of *Tlr11* knock-out (*Tlr11*^*-/-*^) RAW264.7 cells (using primers TLR11-F and TLR11-R) were sequenced and compared with the published mouse *Tlr11* gene by BLAST (https://blast.ncbi.nlm.nih.gov/Blast.cgi). This *Tlr11*^*-/-*^ RAW264.7 cell line has a 243 bp deletion in exon 2 of *Tlr11* and several frame-shift mutations.
(TIF)

S3 Fig Amino acid sequence alignment between *T*. *gondii* MIC3 peptide and *T*. *gondii* profilin-like protein. Software Bioedit was used to compare the amino acid sequences of *T*. *gondii* MIC3 peptide and profilin-like protein.
(TIF)

S4 Fig Protein structure simulation of *T*. *gondii* MIC3 peptide. SWISS-MODEL (https://swissmodel.expasy.org/) was used to simulate the tertiary structure of MIC3 peptide. The model validation parameters are as follows: MolProbity Score 2.54, Clash Score 3.81, Ramachandran Favoured 77.78%.
(TIF)

S1 Text Original images for Western blotting. (DOCX)

S1 Data Original values used to build graphs. (XLSX)
