# Supplementary material for: Toxoplasma gondii microneme protein MIC3 induces macrophage TNF-α production and Ly6C expression via TLR11/MyD88 pathway
Source: PLoS Negl Trop Dis. 2023 Feb 2;17(2):e0011105. doi: 10.1371/journal.pntd.0011105 (PMC9928027; doi:10.1371/journal.pntd.0011105)
Supplement: S1 Table — (DOCX) [file pntd.0011105.s001.docx]

**S1 Table**

**Primers used in this study.**

| Name | Sequence (5’-3’) |
| --- | --- |
| Mus-GAPDH-F | AGGTCGGTGTGAACGGATTTG |
| Mus-GAPDH-R | TGTAGACCATGTAGTTGAGGTCA |
| Mus-TNF-α-F | AAGCCTGTAGCCCACGTCGTA |
| Mus-TNF-α-R | GGCACCACTAGTTGGTTGTCTTTG |
| Mus-IL-6-F | GAGGATACCACTCCCAACAGACC |
| Mus-IL-6-R | AAGTGCATCATCGTTGTTCATACA |
| Mus-IL-10-F | GACCAGCTGGACAACATACTGCTAA |
| Mus-IL-10-R | GATAAGGCTTGGCAACCCAAGTAA |
| Mus-iNOS-F | GGAGCGAGTTGTGGATTGTC |
| Mus-iNOS-R | GTGAGGGCTTGGCTGAGTGAG |
| Mus-Arg-1-F | CAGAAGAATGGAAGAGTCAG |
| Mus-Arg-1-R | CAGATATGCAGGGAGTCACC |
| TLR11-F | CTACCTGCTGGAGTCACACATC |
| TLR11-R | AAACTTAGGCAACAGGGAGCAT |
| Tlr11-qPCR-F | ATATTGCCTGACTTATAGCC |
| Tlr11-qPCR-R | AAGGTCCTGAAACTTTGTTA |
| Tlr11-qPCR-N-F | TGCCCAGAAATACAGAGG |
| Tlr11-qPCR-N-R | GGAGTGGAAGCGAATAAA |
| Homo-GAPDH-F | TATGACAACAGCCTCAAGAT |
| Homo-GAPDH-R | AGTCCTTCCACGATACCA |
| Homo-TNF-α-F | GCCTGTACCTCATCTACTC |
| Homo-TNF-α-R | CCTTGGTCTGGTAGGAGA |
| Homo-IL-6-F | AAGTCCTGATCCAGTTCCT |
| Homo-IL-6-R | GCAGAATGAGATGAGTTGTC |
| Homo-IL-10-F | TGGAGCAGGTGAAGAATG |
| Homo-IL-10-R | GTAGGCTTCTATGTAGTTGATG |
| Homo-iNOS-F | GCCTCGCTCTGGAAAGA |
| Homo-iNOS-R | TCCATGCAGACAACCTT |
| Homo-Arg-1-F | AGTCAAGAAGAACGGAAGAA |
| Homo-Arg-1-R | GTGGTTGTCAGTGGAGTG |
