# Supplementary material for: Toxoplasma gondii microneme protein MIC3 induces macrophage TNF-α production and Ly6C expression via TLR11/MyD88 pathway
Source: PLoS Negl Trop Dis. 2023 Feb 2;17(2):e0011105. doi: 10.1371/journal.pntd.0011105 (PMC9928027; doi:10.1371/journal.pntd.0011105)
Supplement: S2 Table — (DOCX) [file pntd.0011105.s002.docx]

**S2 Table**

***Ct* values of *Tlr11* in wild-type and *Tlr11^-/-^* RAW264.7 cells.**

| **Cell lines** | ***Ct* values of *Tlr11* ^a^** |
| --- | --- |
| *wild-type* RAW264.7 | 28.75 |
|  | 28.01 |
|  | 29.09 |
|  | 28.38 |
| *Tlr11^-/-^* RAW264.7 | undetected |
|  | undetected |
|  | 36.63 |
|  | undetected |

a: Primers Tlr11-qPCR-N-F and Tlr11-qPCR-N-R were used for qPCR.
