# Supplementary figures and images for: Toxoplasma gondii microneme protein MIC3 induces macrophage TNF-α production and Ly6C expression via TLR11/MyD88 pathway
Source: PLoS Negl Trop Dis. 2023 Feb 2;17(2):e0011105. doi: 10.1371/journal.pntd.0011105 (PMC9928027; doi:10.1371/journal.pntd.0011105)

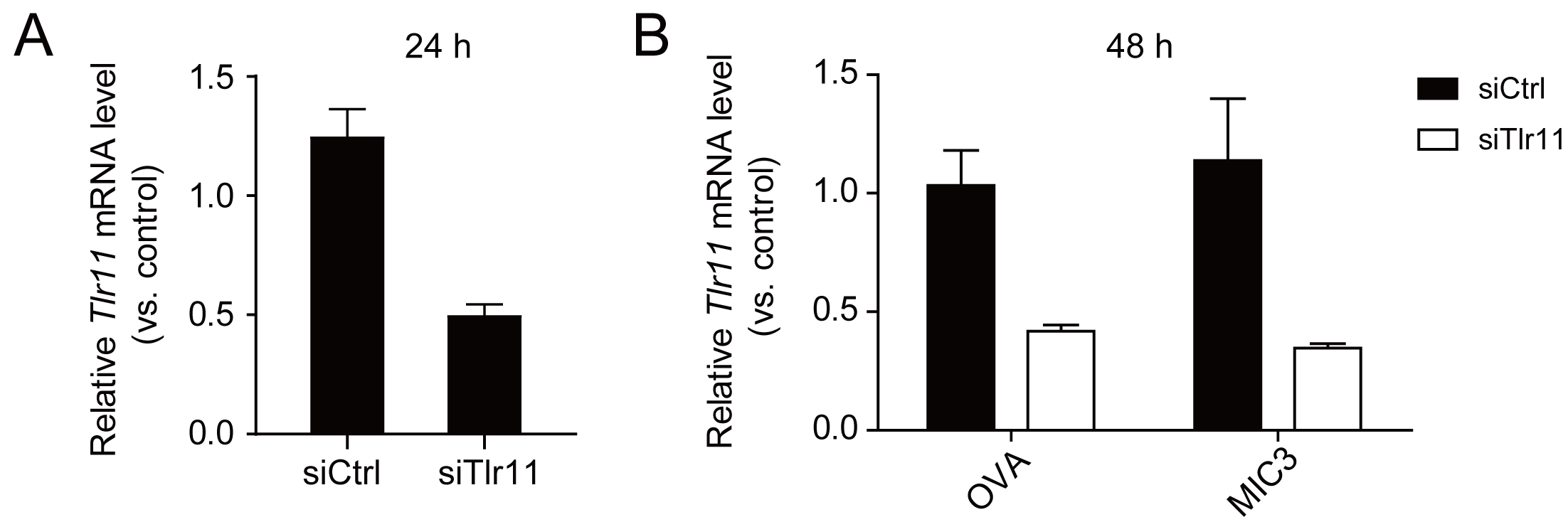

Supplement: S1 Fig — Tlr11 knock-down efficiency in RAW264.7 cells 24 h (A) and 48 h (B) after transfection. Tlr11 expression levels were knocked-down by siRNA targeting Tlr11 (siTlr11). A control siRNA (siCtrl) that targets a scrambled sequence were used as control. 24 h after transfection, RAW264.7 cells were treated with 4 μg/ml MIC3 and OVA for another 24 h. The mRNA levels of Tlr11 and Gapdh were measured 24 h and 48 h after siRNA transfection using qRT-PCR. 1 = no change. Each bar indicates the mean value ± S.D. (n = 3 independent replicates). (TIF) [file pntd.0011105.s003.tif]

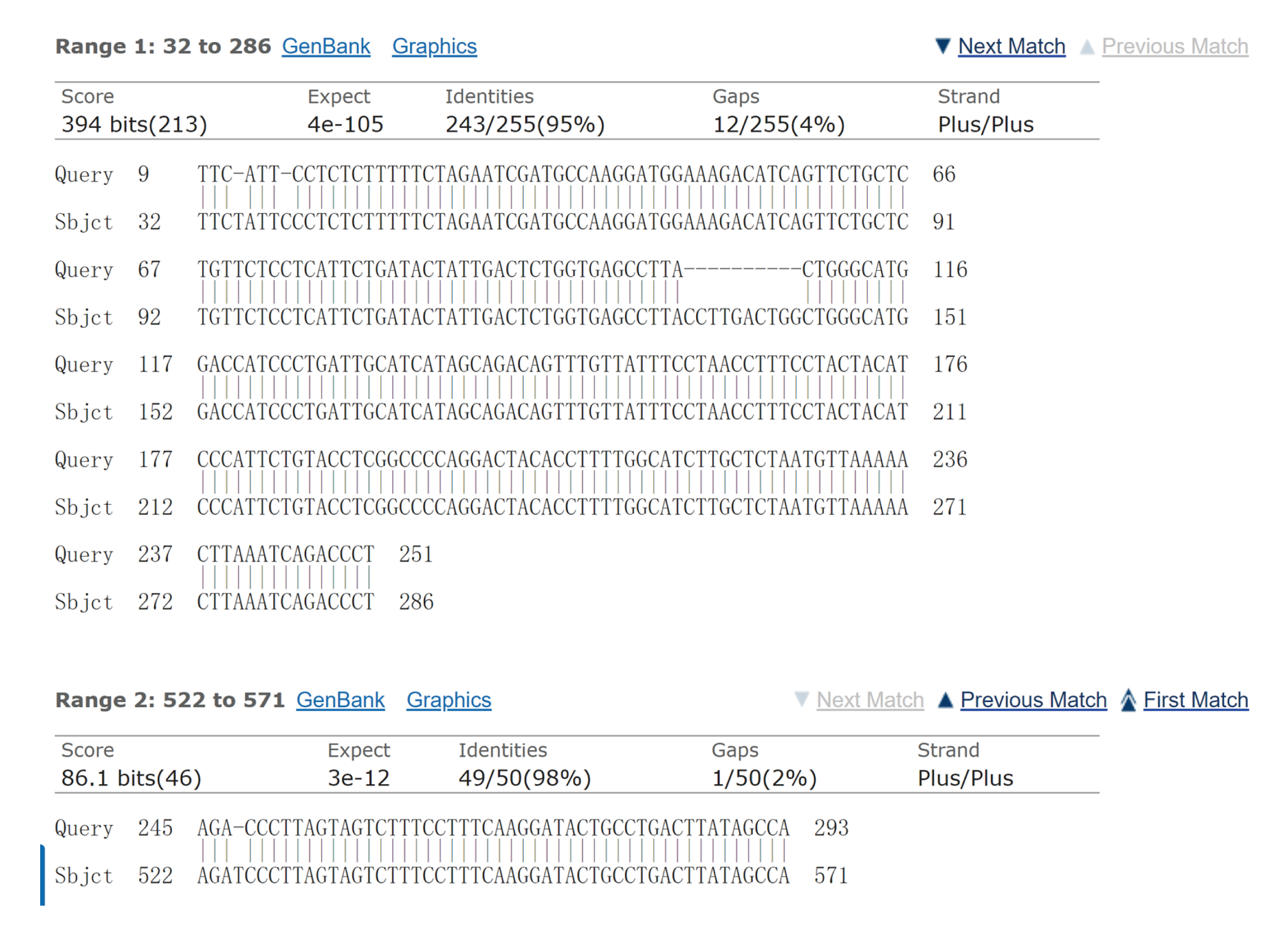

Supplement: S2 Fig — The PCR products of Tlr11 knock-out (Tlr11-/-) RAW264.7 cells (using primers TLR11-F and TLR11-R) were sequenced and compared with the published mouse Tlr11 gene by BLAST (https://blast.ncbi.nlm.nih.gov/Blast.cgi). This Tlr11-/- RAW264.7 cell line has a 243 bp deletion in exon 2 of Tlr11 and several frame-shift mutations. (TIF) [file pntd.0011105.s004.tif]

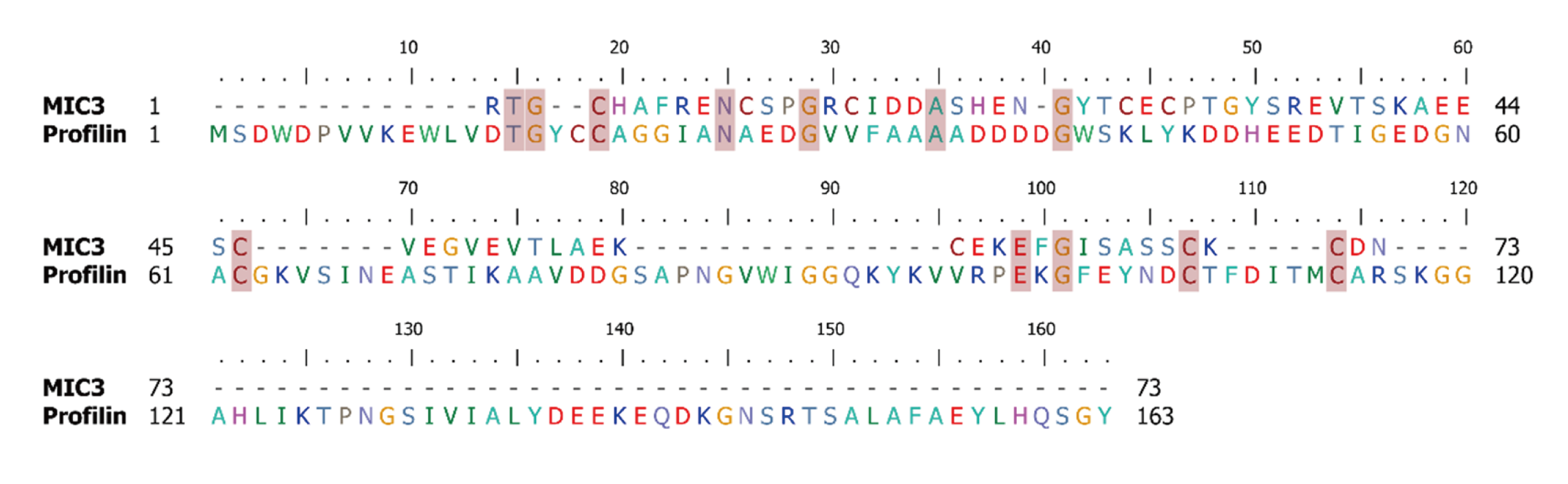

Supplement: S3 Fig — Software Bioedit was used to compare the amino acid sequences of T. gondii MIC3 peptide and profilin-like protein. (TIF) [file pntd.0011105.s005.tif]

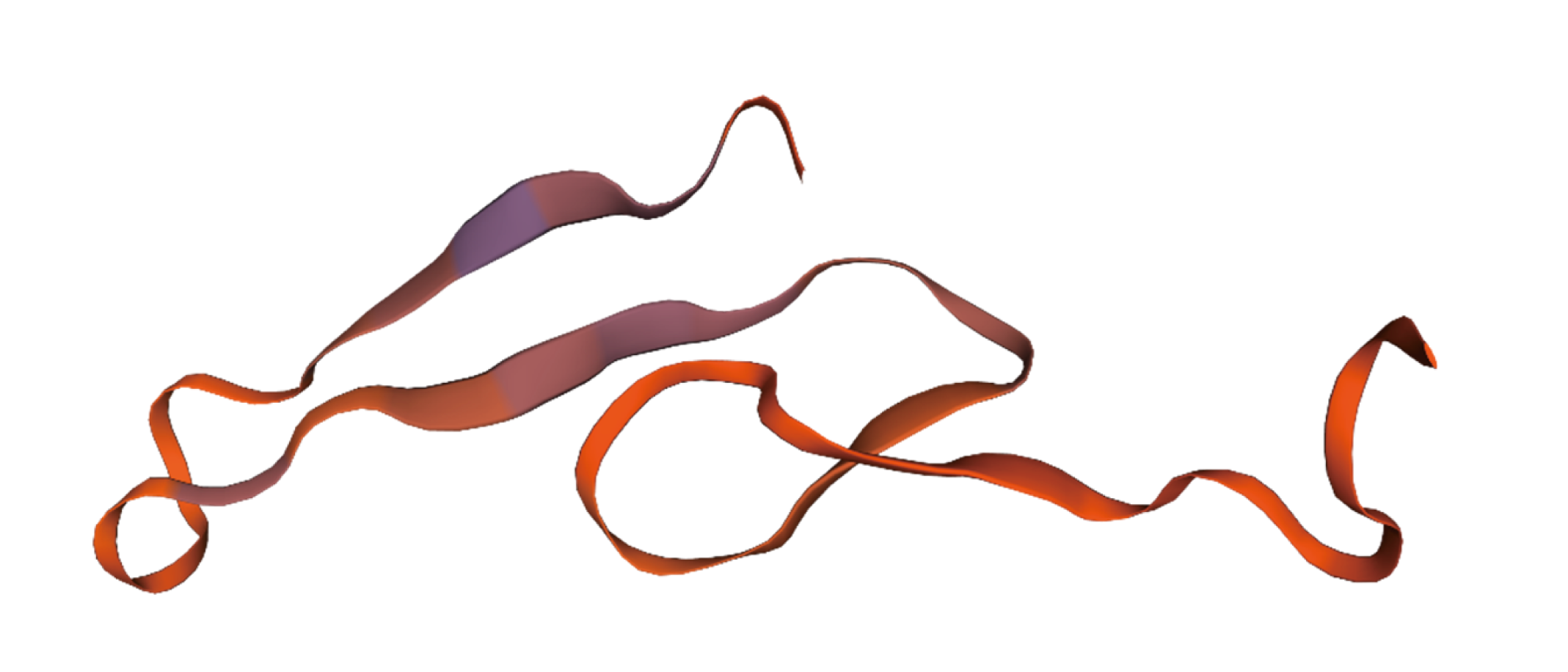

Supplement: S4 Fig — SWISS-MODEL (https://swissmodel.expasy.org/) was used to simulate the tertiary structure of MIC3 peptide. The model validation parameters are as follows: MolProbity Score 2.54, Clash Score 3.81, Ramachandran Favoured 77.78%. (TIF) [file pntd.0011105.s006.tif]
