# Supplementary material for: Toxoplasma gondii microneme protein MIC3 induces macrophage TNF-α production and Ly6C expression via TLR11/MyD88 pathway
Source: PLoS Negl Trop Dis. 2023 Feb 2;17(2):e0011105. doi: 10.1371/journal.pntd.0011105 (PMC9928027; doi:10.1371/journal.pntd.0011105)

**S1 Text. Original images for Western blotting.**

**Original images for Fig 1B**

p-NF-κB


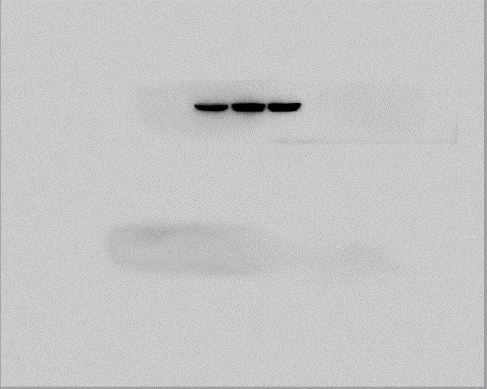


GAPDH


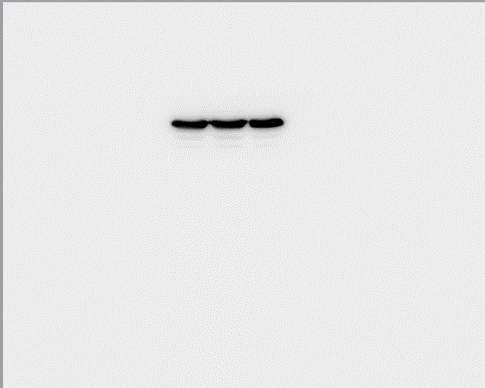


**Original images for Fig 3G**

p-NF-κB


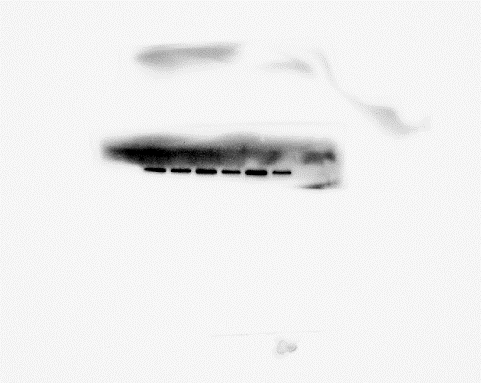


GAPDH


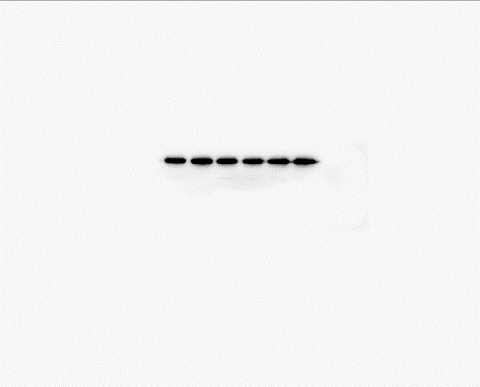


**Original images for Fig 5F**

p-NF-κB

Wild-type RAW264.7 cells Tlr11^-/-^ (*Tlr11* knock-out) RAW264.7 cells


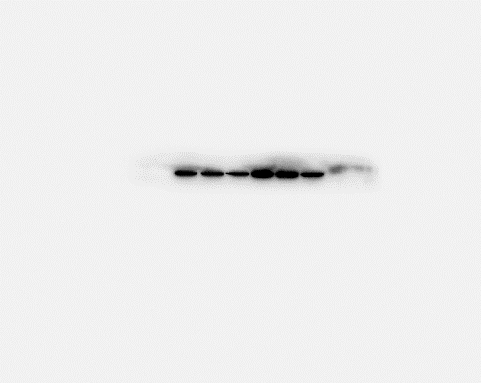

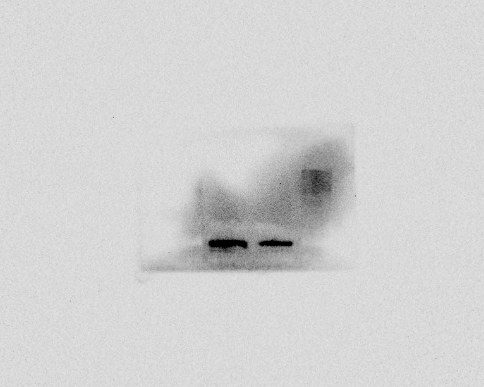


GAPDH

Wild-type RAW264.7 cells Tlr11^-/-^ (*Tlr11* knock-out) RAW264.7 cells


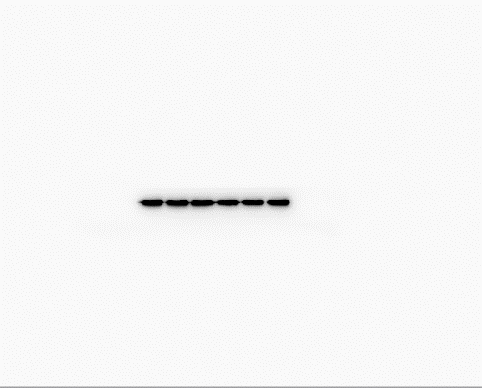

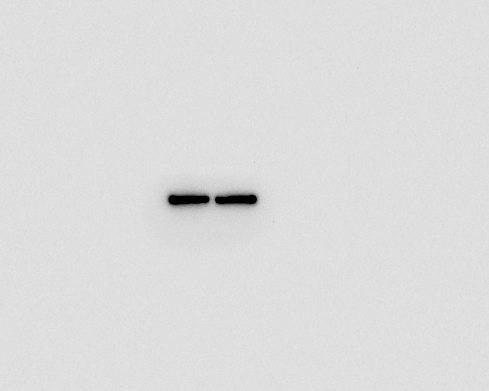


**Original images for Fig 6C**

p-NF-κB


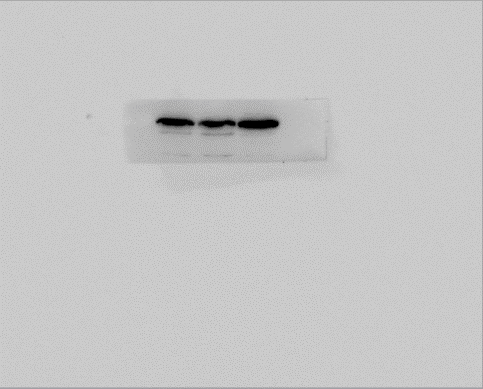


GAPDH


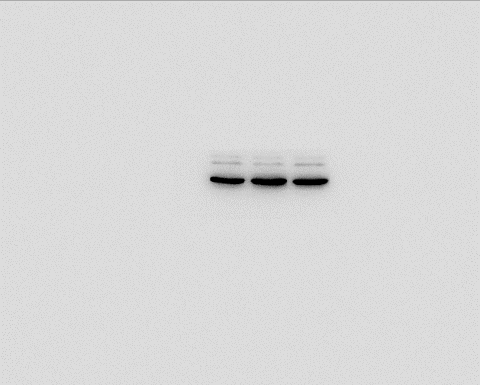

Supplement: S1 Text — (DOCX) [file pntd.0011105.s007.docx]
